# 

**Published:** 1892-10

**Authors:** 


WHOLE NUMBER,
CCCLXXIII.

New Series.

October, 1892.

VOL. XXXII.
No. 3.

### BUFFALO MEDICAL AND SURGICAL JOURNAL.

Established 1845 by AUSTIN FLINT, M. D.

**Editors:**

THOS. LOTHROP, M. D.    WM. WARREN POTTER, M. D.

**Associate Editors:**

WILLIAM C. KRAUSS, M. D.    JAMES WRIGHT PUTNAM, M. D.

JOHN A. MILLER, Ph. D.    DeLANCEY ROCHESTER, M. D.

JOHN PARMENTER, M. D.

SCRIPTA FERUNT ANNOS

EUROPEAN AGENCY,
TRÜBNER & CO.,
57 AND 59 LUDGATE HILL, LONDON, E. C.

BUFFALO:
1892.

FOREIGN
SUBSCRIPTION, $2.50

Entered at the Post-Office at Buffalo, N. Y., as Second-class Mail Matter.

*Times Printing House, Buffalo, N. Y.*

SUBSCRIPTION PRICE, $2.00 IF PAID IN ADVANCE; OTHERWISE $2.50 A YEAR.

PUBLISHED MONTHLY.

Table of Contents on Page V

